# Molecular model linking Th2 polarized M2 tumour‐associated macrophages with deaminase‐mediated cancer progression mutation signatures

**DOI:** 10.1111/sji.12760

**Published:** 2019-03-18

**Authors:** Jared Mamrot, Siddharth Balachandran, Edward J. Steele, Robyn A. Lindley

**Affiliations:** ^1^ GMDxCo Pty Ltd Melbourne Victoria Australia; ^2^ Blood Cell Development and Function Program Fox Chase Cancer Center Philadelphia Pennsylvania; ^3^ CYO’Connor ERADE Village Foundation Perth Western Australia Australia; ^4^ Melville Analytics Pty Ltd Melbourne Victoria Australia; ^5^ Faculty of Medicine, Dentistry & Health Sciences, Department of Clinical Pathology University of Melbourne Melbourne Victoria Australia

**Keywords:** cancer progression, combinatorial association, cytosine and adenosine deaminases, extracellular vesicles, tumour‐associated macrophages

## Abstract

A new and diverse range of somatic mutation signatures are observed in late‐stage cancers, but the underlying reasons are not fully understood. We advance a “combinatorial association model” for deaminase binding domain (DBD) diversification to explain the generation of previously observed cancer‐progression associated mutation signatures. We also propose that changes in the polarization of tumour‐associated macrophages (TAMs) are accompanied by the expression of deaminases with a new and diverse range of DBDs, and thus accounting for the generation of new somatic mutation signatures. The mechanism proposed is molecularly reminiscent of combinatorial association of heavy (H) and light (L) protein chains following V(D)J recombination of immunoglobulin molecules (and similarly for protein chains in heterodimers α/β and γ/δ of V(D)Js of T Cell Receptors) required for pathogen antigen recognition by B cells and T cells, respectively. We also discuss whether extracellular vesicles (EVs) emanating from tumour enhancing M2‐polarized macrophages represent a likely source of the de novo deaminase DBDs. We conclude that M2‐polarized macrophages extruding EVs loaded with deaminase proteins or deaminase‐specific transcription/translation regulatory factors and like information may directly trigger deaminase diversification within cancer cells, and thus account for the many new somatic mutation signatures that are indicative of cancer progression. This hypothesis now has a plausible evidentiary base, and it is worth direct testing in future investigations. A long‐term objective would be to identify molecular biomarkers predicting cancer progression (or metastatic disease) and to support the development of new drug targets before metastatic pathways are activated.

## 1 DEAMINASES AND CANCER

Innate immune and Inflammatory responses to infection^1^, ^2^ trigger the activation of interferon‐stimulated Gene (ISG) pathways.^3^, ^4^, ^5^ Among the hundreds of anti‐pathogen ISG products co‐ordinately expressed during innate immune and inflammatory responses across cell types are the AID/APOBEC family of Cytosine (C‐site) and ADAR family of Adenosine (A‐site) deaminases.^3^, ^4^, ^6^, ^7^ The cytidine deaminases of AID and the APOBECs cause C‐to‐U(T) transition mutations (and set up downstream C‐to‐A, C‐to‐G transversion mutations) and act on single‐stranded DNA. The ADARs, or adenosine targeting deaminases causing A‐to‐I(G) transition mutations (and set up downstream A‐to‐T and A‐to‐C transversion mutations), and target nascent double‐stranded RNA stem‐loop substrates,^8^ or RNA:DNA hybrids.^9^ These DNA or RNA conformations occur during both cellular and viral transcription.^10^, ^11^

Whilst the antiviral functions of these deaminases are well studied, it has also been established that genomic mutations of the host cell occur as “collateral damage” and can accumulate from one cell generation to the next when such anti‐pathogen deaminase responses become dysregulated. Ultimately, the accumulation of deaminase mutations during transcription may lead to tumorigenesis.^10^, ^12^, ^13^

In 2010, we showed that many non‐lymphoid cancers display the same strand‐biased spectrum of point mutations at G:C and A:T base pairs in non‐Ig genes as observed in normal physiological Ig somatic hypermutation (SHM) of B cells, indicative of AID and ADAR1 action during transcription.^14^, ^15^ Later, we demonstrated the existence of motifs known to be associated with both AID/APOBECs and ADAR deaminases in non‐Ig genes of uninfected somatic cells.^10^ Also in 2013, Burns et al^16^ showed that APOBEC3B enzymatic action may serve as a source of mutations in breast cancer. In our work, the “telltale” deaminase‐linked targeted somatic mutation (TSM) signatures were identified for a large proportion of both cytidine and adenosine deaminases. Deaminase targeting was shown to be specified by the dominant mutation type (eg, G > A), the target nucleotide motif (eg, RGYW, R = A/G, Y = C/T, W = T/A) and the target nucleotide site within the codon reading frame (eg, MC2, referring to the mutated codon second nucleotide site, read in the 5‐prime to 3‐prime direction). Target motifs in a TSM signature therefore define the inferred deaminase binding domain (Inf‐DBD) of each unique deaminase “binding state.” In reporting these early TSM results, it was also shown that the mechanisms involved a process to discriminate between the cytosines on the “top” or non‐transcribed strand (by convention read as mutations of “C”), and those on the “bottom” or transcribed strand (by convention read as mutations of “G”).^10^

As further support for the TSM‐profiling approach briefly described here, the analytical methods involved have also been applied to the analysis of the dbSNP database of curated clinically significant single nucleotide polymorphisms (SNPs). It was shown that a significant number of human SNPs appear to have arisen by AID/APOBEC and ADAR deaminations leading to the germline incorporation of these SNPs over evolutionary time‐scales.^17^ Another TSM application has been used to show that the range of “spontaneous” mutations arising in RNA (and DNA) viral genomes early in acute phases of Flavivirus (eg, ZIKV, HCV) and Hepadnavirus (eg, HBV) infections are due to the viral RNA‐dependent RNA polymerase replicases incorporating AID/APOBEC and ADAR‐mediated deaminations as transition mutations into viral progeny genomes.^18^

Thus, there is mounting evidence supporting the view that TSM signatures observed in DNA are highly targeted, and manifest as somatic mutations that accumulate in the host cell genome as collateral damage during an innate immune response to a pathogenic insult. The resulting de novo somatic mutation patterns are now widely known to be associated with cancer progression.^13^

## 2 DEAMINASES AND CANCER PROGRESSION

The rapid maturation of AID/APOBEC and ADAR deamination mutation signatures associated with a prediction of cancer progression was first shown by analyzing changes in the Inf‐DBDs of high‐grade serous ovarian (HGsOv) carcinoma. A number of new deaminase‐linked TSM signatures and their association with disease progression were identified.^13^ These are referred to as cancer‐progression associated signatures (C‐PAS). The C‐PAS were identified as uncorrected de novo somatic mutations using whole exome sequencing (WES). Each C‐PAS is defined as a possible TSM signature variant—or heterodimer—of the DBD homodimer for each of the key deaminases. It was deduced that the cancer seems to “throw a switch” to a new level of maturation: The result is the generation of new DBDs that may “predict” cancer progression. The C‐PAS may therefore be interpreted as a part of a much larger set of non‐canonical C‐site and A‐site motifs. A key question we focus on is: How is the “maturation” achieved at the level of Inf‐DBDs in AID/APOBEC and ADAR deaminase proteins?

In addition to the above genomic analyses, there are other plausible molecular explanations for generating the functional maturation of C‐to‐U(T) and A‐to‐I(G) deaminase DBD diversity in late‐stage cancer. Additional diversity has previously been contributed by ADARs self‐editing their active DBDs, and thereby altering their function,^19^ and that result in changes in the deaminase “motifs” targeted.^20^ Such variants may exhibit the potential for alternative ADAR targeting preferences during tumour progression. A further factor to consider in ADAR‐dependent cancer progression is the ADAR1 and ADAR2 RNA editing imbalances previously observed in cancer. ADAR1 overexpression acts as an oncogene or tumour promoter, and ADAR2 has been found to act as a tumour suppressor in both hepatocellular carcinoma and gastric cancer.^21^, ^22^ TP53 is also known to regulate A/T‐focused (and ADAR related) mutations in normal physiological Ig SHM.^23^ Thus, it is logical to speculate that the mechanism by which this occurs might be a TP53‐related switch: It has been postulated that when a critical mass of genomic damage is sensed by TP53, the “upregulation” of APOBEC3 family transcription and expression may be triggered.^24^ Given the current thought in the deaminase field, this TP53‐link to regulation of the tandemly arrayed APOBEC3 genes is consistent with upregulation of the endogenous APOBEC3s in the cancer cells themselves possibly as the drivers of the early stages of mutation. However, it is the source of this “upregulation” in the late stages of cancer that we are concerned with here.

With these genomic deaminase mutator mechanisms firmly in mind, we now discuss a general model for cancer progression based on deaminase‐mediated Innate Immunity via Functional Combinatorial Association (shown in Table 1).

**Table 1:** A model for AID/APOBEC and ADAR heterodimer formation in advanced diseases

	Chronic diseases & cancer progression	
	Maturation of genomic mutagenesis	
	Early	Late
Immune state	Innate	Innate, Adaptive
Deaminase DBD state	Homodimers	Heterodimers
AID	1	10
APOBEC1	1	10
APOBEC2	1	10
APOBEC3A	1	10
APOBEC3B	1	10
APOBEC3C	1	10
APOBEC3D	1	10
APOBEC3F	1	10
APOBEC3G	1	10
APOBEC3H	1	10
APOBEC4	1	10
	Total = 11 DBDs	Total = 110 potential DBDs
ADAR1p150 1200 aa	1	5
ADAR1p110 931 aa	1	5
ADAR2 701 aa	1	5
ADAR2 674 aa	1	5
ADAR2 741 aa	1	5
ADAR2 714 aa	1	5
	Total = 6 DBDs	Total = 30 potential DBDs
ADAR3	1 (blocker brain)	?

DBD, deaminase binding domain.

The number of potential heterodimers with novel C‐site specific DBDs under a random combinatorial protein association model is shown for the AID/APOBEC gene family (from homologous sequences documented in Smith et al^6^); the number of potential novel A‐site specific DBDs under a random combinatorial association model for the common isoforms ADAR1/2 group of similar sequences (Samuel^7^).

### 2.1 A combinatorial association model to explain DBD diversification

We advance a functional “Combinatorial Association Model” to explain the generation of APOBEC DBD diversity. Formulation of the computational model is based on the assumption of DBD shuffling and chimera (heterodimer) formation as a result of the molecular mechanisms described in the previous section and reviewed in Lindley et al.^13^

Table 1 shows one approach to the estimation of the possible number of heterodimers for each DBD state. In the AID/APOBEC group,^6^ the number of potential DBDs under a random Combinatorial Association Model is 11 × 11 (broken down into homodimers and hypothetical heterodimers in Table 1). For the ADAR1/2 group,^7^ it is potentially 6 × 6 as shown. As cancer progresses, the model predicts a second‐ or late‐stage rapid diversification of the number of potential new target sites in the cancer genome. These sites may be mutated by a new and more diverse range of new deaminase heterodimers. We suggest that the normal tissue expression of the different AID and APOBEC deaminases^25^ is quantifiably altered during chronic pathogen infections (eg, HBV) and during cancer progression, such that expression across all members of the APOBEC3 tandemly arrayed haplotype family (as well as in AID, APOBEC1, APOBEC2, APOBEC4) is elevated leading to new functional combinatorial associations at the protein level of different zinc DBDs. This concept was briefly discussed in Lindley et al.^13^

For APOBEC1, APOBEC2, APOBEC4 and the APOBEC3 family series of deaminases,^6^, ^26^ homodimer formation is considered the norm in healthy tissues and in acute in vitro stimulations. We hypothesize that during DBD maturation of specificity, different DBD heterodimer combinations are possible (A3A/AID, A3A/A1, A3A/A2, etc) and may be potentially created (Table 1). In a polymorphic heterozygote, and with the known tendency to multimerization,^27^ the potential number of novel combinatorial DBD associations could be even greater in a given cancer environment. However, it is important to note that the number of possible heterodimers that will result in a successful new DBD for each deaminase is further reduced by selection because not all potential heterodimers are expected to successfully bind to their respective ssDNA target sites during transcription.

There are also logically fewer potential ADAR protein heterodimers^7^ under the same random association model (Table 1). It is conceivable that the same expansion of functional combinatorial DBD diversification takes place for ADAR1 and ADAR2 isoforms causing diverse A‐to‐I targeting specificities at many non‐canonical A‐sites on dsRNA and hybrid RNA:DNA substrates. For the AID/APOBECs, the heterodimers in advanced infection and diseased states may also show a composition bias as reflective of the normal dominant APOBEC variant expression in that tissue. For example, in blood and lung where APOBEC3A is normally dominantly expressed,^25^ the heterodimers are hypothesized to be primarily combinations of A3A with other AID/APOBEC members (ie, A3A/AID, A3A/A1, A3A/A2). The exact composition may reflect the relative expression levels of the other AID/APOBEC proteins, and thus protein concentrations in given assembly vesicles.

## 3 COULD TUMOUR‐ASSOCIATED MACROPHAGES BE THE SOURCE OF DEAMINASES?

The activated macrophage cell type, and its associated functions, is the first line of defence against pathogen infections in all eukaryotic and prokaryotic organisms.^1^, ^2^, ^28^ The conserved evolutionary functions of the “macrophage” are found in primitive single cell and free‐living amoeba and the early metazoan slime moulds, with their alternating single cell and multicellular reproductive life cycles. Thus, the cells of the monocyte‐derived macrophage series in vertebrate blood streams and lymphatics deploy ancient “pathogen kill” and “cellular repair” strategies that are highly conserved. The EVs secreted include hundreds, if not thousands, of different proteins and other molecular species in their cargos. Yet, evidence in the literature for AID/APOBEC and ADAR deaminases within the secreted extracellular vesicles (EVs) from stimulated tumour‐associated macrophages (TAMs) remains limited.

Activated macrophages are the main cell types responsible for effective first line cell‐mediated immunity against intracellular infection^29^ and tumour immunity.^30^ The initial infection triggers the Innate Immune functions of the ISG cascade in all cells, which includes expression of AID/APOBEC and ADAR deaminases.^3^, ^4^ This is particularly relevant to the activated monocyte‐derived macrophage cell series, and cytosolic vesicles (P bodies) of high molecular mass aggregates of presumed APOBEC3G monomers are well documented.^6^, ^27^, ^31^ Whilst there are few, if any, EV peptide/proteome studies reporting ADAR1/2 deaminase protein content, there are many studies reporting clear evidence of APOBEC3 proteins within EVs. For example, Cypryk et al^32^ report EVs with APOBEC3A and APOBEC3C proteins from influenza A virus infected macrophages. However, similar studies with fungal β‐glucan stimulation showed no incorporation of APOBEC3 proteins.^33^ Human cell lines infected with HIV‐1 have been shown to secrete EVs containing active APOBEC3G proteins^34^ that confer resistance to HIV on transfer to recipient cells. In further studies from the same group, APOBEC3G and APOBEC3H were found to be secreted in stimulated human cells in EVs which inhibited LINE‐1 and Alu retrotransposition in target cells.^35^ More recently, Reales‐Calderon et al^36^ have shown that *Candida albicans* stimulation of human THP‐1 macrophages produces EVs containing APOBEC3C. It should also be noted that APOBEC proteins were not found in EVs secreted by TAMS in two other recent studies.^37^, ^38^

It is also known that in some specific cases, transfer of Innate Immune regulatory states can occur via EVs. EVs derived from neural stem cells have been shown to transfer IFN‐γ, activating the ISG pathway via Stat1 signalling in target cells.^39^ Microvesicles secreted by macrophages have also been shown to shuttle invasion‐potentiating microRNAs into breast cancer cells.^40^ TAMS‐derived miR‐21 has been shown to confer cisplatin resistance in gastric cancer cells,^41^ and TAMS‐derived exosomes are known to promote migration of gastric cancer cells by EV transfer of functional Apolipoprotein E.^42^ In respect to HCV infection, Cai et al^43^ have shown macrophage‐derived EVs induce long‐lasting immunity.

There is therefore great potential for M1/M2 TAMs to secrete various stimulators of ISG pathways and thus directly affect both activity and gene expression of the APOBEC and ADAR family proteins in target cancer cells.^44^ The M2 state (anti‐inflammatory state) may also directly activate elevated “deaminase binding domain shuffling” (per Table 1), generating functional protein heterodimers during the second stage of cancer progression (metastasis). This would produce a more diverse set of functional DBDs targeting additional sites of the cancer genome. This could be exploited by the host to promote the death of cancer cells by mutational overload, increase neo‐epitope production, and thus to make the cancer cells more susceptible to immunotherapy.

Of special relevance here, the “inhibit and kill” functions of the macrophage through the induction by the nitrous oxide (NO) metabolic programme and the arginine/ornithine “healing” or wound repair pathways have been identified with the two alternative polarized states of “M1” and “M2” macrophage phenotypes.^2^, ^45^, ^46^, ^47^ Thus, within this combatant tumour‐host microenvironment, the macrophage plays a pivotal role in the progression of the cancer. Macrophages do this both through their own plasticity driving inhibition (M1) and/or encouragement of tumour growth (M2, wound repair pathways of angiogenesis for example). The tumour‐enhancing functions of M2 TAMS have been demonstrated by Jeff Pollard and colleagues.^45^, ^46^ Recruited monocytes‐macrophages arriving at the tumour site in the M1 polarized state appear to convert rapidly to M2.^47^ In quintessential cases of progressing high‐grade serous ovarian (HGsOv) cancer, a favourable clinical prognosis depends on a high M1/M2 ratio within the local tumour microenvironment (TME) itself and the close physical conjunction of macrophages and tumour cells.^38^ A future clinical goal is to exploit this known macrophage functional plasticity^47^ by implementing procedures involving cytokine and chemokine signalling to convert M2 cells into inhibitory M1 cells or moderate signals and functions secreted by M2 which influence the TME. That is, just as there is evidence suggesting that there is a degree of plasticity associated with the formation of new deaminase heterodimers as cancer progresses (as previously discussed), there is also evidence that the TME exhibits a complex mix of macrophage plasticity and activation states associated with cancer progression (M1<‐>M2).

Here we interrogate the relationship between the events triggering the formation of new deaminase heterodimers and the changes in the polarized states of M1 and M2 as cancer progresses. To address this, we will also need to consider the role of lymphocyte activation in the tumour microenvironment.

## 4 LYMPHOCYTE ACTIVATION AND THE HOST‐TUMOUR RELATIONSHIP

The basic model of lymphocyte activation is also fundamental to our current understanding of immunity and the host‐tumour relationship as cancer progresses. Antigen‐specific adaptive immunity and the corollary development of our understanding of T cell‐mediated regulation of the tumour‐host relationship led by Bretscher^48^, ^49^, ^50^ incorporate the additional functional classifications in T helper (Th) cell functions in Th1 and Th2 T lymphocyte lineages.^51^ Although these antigen‐specific T cell responses are secondary to the central role of the macrophage/dendritic cell/follicular dendritic cell lineages in immunity, these cells are important as they regulate the cytokine environment and they present antigens to further activate and regulate adaptive T and B lymphocyte immunity in the tumour microenvironment. It has been known for over a decade that the result is a direct correspondence, at the level of the functional cytokine mixes involved, between the M1 and M2 macrophage polarization and the Th1 and Th2 phenotypes of T cell antigen‐specific regulation.^28^ That is, there is a striking correlation between the M1 macrophage phenotype and Th1 lymphocytes (M1/Th1), and the M2 macrophage phenotype and Th2 lymphocytes (M2/Th2).

## 5 COMMUNICATION BETWEEN CELLS IN THE TME

Communication between cells is necessary to co‐ordinate and regulate deaminase and macrophage polarization changes in the TME. It is known that cytosolic proteins, lipids, carbohydrates, small regulators and RNAs (including mRNAs and miRNAs) typical of the functional and regulatory cytosolic state of a cell do communicate with neighbouring cells, or more distant cells throughout the vascular and lymphatic systems. This occurs via their EVs.^44^, ^52^ The budding of viral particles is related to this process, which can also transfer cytosolic cargos and antiviral deaminases (eg, the widely studied antiviral deaminase APOBEC3G) in EVs.^53^, ^54^ Moreover, within the inflammatory‐focused tumour microenvironment, EV communication can occur in principle in both directions from activated M1/M2 macrophages^32^, ^33^, ^36^, ^37^, ^40^, ^41^, ^42^ and from tumour cells to macrophages themselves.^56^, ^57^, ^58^, ^59^, ^60^

## 6 MOLECULAR MODEL LINKING M2 TAMs WITH DEAMINASE‐MEDIATED CANCER PROGRESSION

**Figure 1:**
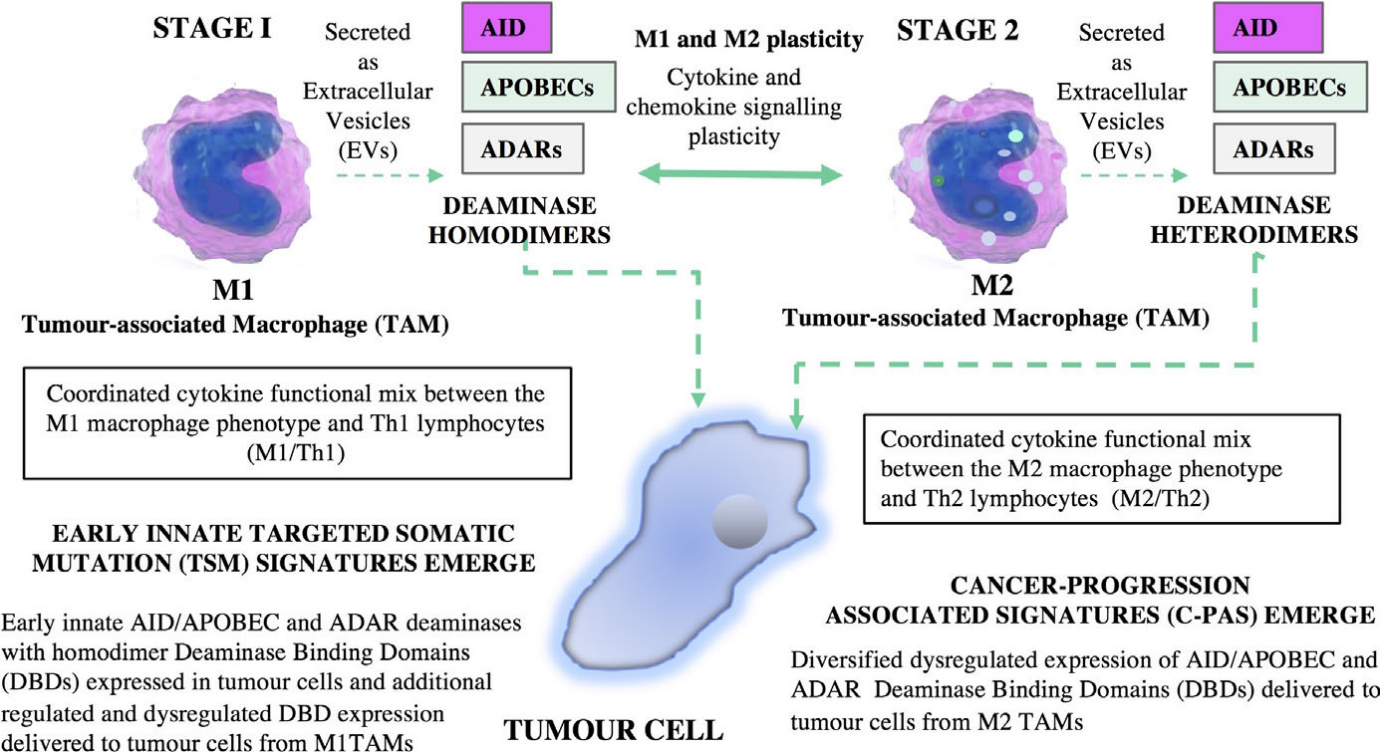
A model for Deaminase‐Mediated Innate Immunity via Functional Combinatorial Association for cancer progression. The macrophages are the central players in directing, regulating and executing key functions involving changes in deaminase activity as cancer progresses

Thus, in oncogenesis, the collective processes involve an ongoing and classical “armed stand‐off” between host immunity, tumour mutagenesis and adaptive survival. This involves an orchestrated set of molecular processes that appear to occur in two stages: The first involves pre‐ or early‐stage cancer development where the M1/M2 ratio is high, and most of the TSM signatures are identified as the main homodimers for key deaminases. The second stage is characterized by a low M1/M2 ratio, and C‐PAS can be identified and are indicative of deaminase heterodimers. The main features of this model for cancer progression are shown in Figure 1.

We propose that M1 and M2 TAMs secrete deaminase proteins (APOBEC, ADAR, AID) that are loaded into extracellular vesicles (EVs) within the pro‐ (M1) and anti‐ (M2) inflammatory tumour microenvironment. Further, we propose that ADAR1 and ADAR2 play essential roles in physiological regulation in situ and are not for promiscuous export, for example the control of inappropriate Innate Immunity cascades to self dsRNA structures, as described by Liddicoat et al.^61^ Via this mechanism, deaminases and other EV contents such as miRNAs and interferons create the conditions in target cells for further dysregulation of deaminase activity that is associated with a higher proportion of polarized M2 TAMs, and a diversification of DBDs that give rise to C‐PAS that are associated with a prediction of progression of disease. This model is also consistent with the tumour‐enhancing roles of M2 TAMS suggested by the work of Pollard and others.^45^, ^46^

Thus, in the proposed model (Figure 1), a cellular subset of the cancers in the M2 TME state are predicted to be associated with a stage‐2 “molecular switch” within the cell that favours “chimeric‐heterodimer” DBDs and elevates mutation levels. We hypothesize that the C‐PAS first documented by Lindley et al^13^ are a new subset of M2 TME induced DBDs that are indicative of high affinity deaminase mutational activity. This mechanism is part of the innate immune process that can potentially eradicate the tumour, or it may provide the tumour with additional immune evasion pathways. Both “direct” and “indirect” models involving AID/APOBEC and ADAR enzymatic activity, and the information transfer triggering the M2 polarization “switch” may be responsible for generating greater DBD diversity in progressing cancer genomes. There is therefore now a need to experimentally establish that macrophages (in particular M2 macrophages) in the tumour microenvironment actually do express and extrude deaminases with new DBDs predicting cancer progression.  Such data would clearly strengthen the model presented here.

Based on this model, we therefore need to view the TME as a combination of aberrant tumour cell death, and a complex mix of macrophage plasticity and activation states (M1↔M2) that are interlinked with endothelial cell activation and deaminase activity. Together, these create an “onco‐regenerative niche” where EVs from dying tumour cells convey information about the tumour cell physiological state to other cells in the microenvironment.^62^ Finally, we also draw attention to the consistency of our hypothetical proposal on tumour immunity and cancer progression with the prior work of the past 40 years by the groups of RJ North and PA Bretscher.^63^, ^64^, ^65^ This type of support from different areas of investigation adds to our analysis. We are proposing here (Table 1, Figure 1) that with time the innate and adaptive immune responses against a cancer evolve from a Th1, M1 mode towards an M2, Th2 mode, and that this evolution results in new genomic mutational signatures predicting cancer progression.

## 7 CONCLUDING REMARKS

The available evidence is consistent with a model in which tumour enhancing M2‐polarized macrophages extrude EVs loaded with deaminase proteins and deaminase‐specific transcription/translation regulatory factors. We believe this directly triggers deaminase DBD diversification within cancer cells, and thus accounts for the observed C‐PAS. This hypothesized deaminase‐mediated cancer progression model thus predicts the presence of an elevated expression of M2 polarized macrophages that is accompanied by the identification of new functionally active APOBEC/ADAR family heterodimers. Future directions include establishing the deaminase content of EVs extruded from M2 TAMS^66^, ^67^ and establishing the dynamic role of active APOBEC (and ADAR) heterodimer genetic markers as common and dominant features predicting cancer progression. Current rapid protein isolation and identification technologies, and genomic sequencing analyses will facilitate future investigation of these hypothesized mechanisms. If successful, genomic tests identifying new C‐PASs associated with molecular markers indicating changes within the TAM environment could be used to predict cancer progression and to guide new drug development strategies for the inactivation of the molecular pathways driving metastatic disease.

## CONFLICT OF INTEREST

The authors declare no conflict of interest.

## AUTHORS’ CONTRIBUTIONS

Robyn A. Lindley conceived of the proposed model and contributed to the writing of the paper. Edward J. Steele, Siddharth Balachandran and Jared Mamrot provided additional insights and also contributed to the writing of the paper.
